# Comparison of the accuracy/precision among guided (static), manual, and dynamic navigation in dental implant surgery: a systematic review and meta-analysis

**DOI:** 10.1007/s10006-025-01462-z

**Published:** 2025-10-10

**Authors:** Filipe Castro, Pedro Pereira, Carlos Falcão-Costa, Artur Falcão, Juliana Campos Hasse Fernandes, Gustavo Vicentis Oliveira Fernandes, José-Vicente Rios

**Affiliations:** 1 Department of Periodontology, University of Seville, Seville, 41009 Spain; 2 FP-I3ID, FCS, Fernando Pessoa University, Porto, 4249-004 Portugal; 3 Private Researcher, St. Louis, MO USA; 4 A.T. Still University - Missouri School of Dentistry & Oral Health, St. Louis, MO USA; 5 Missouri School of Dentistry & Oral Health, A. T. Still University, 1500 Park Dr, St. Louis, 63104 MO USA

**Keywords:** Computer-aided surgery, Dental implants, Dynamic navigation, Accuracy, Guided-implant surgery, Freehand procedure

## Abstract

**Objective:**

To assess whether dynamic navigation (dCAIS) has greater accuracy/precision and less discrepancy in parallelism compared to guided (static, sCAIS) and free-hand (FH) surgery in Implantology.

**Materials and methods:**

A search was conducted across six databases using specific key terms. Randomized controlled trials (RCTs), retrospective or prospective clinical studies published within the last 10 years (2014–2024) were included. Risk of bias was assessed using the Joanna Briggs Institute Critical Appraisal tool. A meta-analysis using a random-effects model was employed. The heterogeneity analysis was conducted using Cochran’s Q-test and Higgins’ I^2^ statistic.

**Results:**

Thirteen articles were included. A total of 554 patients and 687 implants were enrolled, with 215 using the FH system, 195 using sCAIS, and 277 using dCAIS. The meta-analysis compared the following: (1) dCAIS vs. sCAIS; (2) dCAIS vs. FH; (3) sCAIS vs. FH. The first group had a mean difference of -0.08 mm, with a substantial heterogeneity (I² = 52%) and no statistically significant difference (*p* = 0.08); the second presented a mean difference of -0.48 mm, high heterogeneity (I²=89%), and a statistically significant better accuracy for dCAIS than FH (*p* < 0.01); the last comparison found a mean difference of -0.62 mm, with a considerable heterogeneity (I²=84%), and sCAIS showing statistically significantly better accuracy than the FH approach (*p* < 0.01).

**Conclusions:**

Using CAIS (dCAIS or sCAIS) substantially improved accuracy compared to the FH approach, with no statistically significant difference between dCAIS and sCAIS. **Clinical relevance**: The findings support the use of CAIS for improved implant accuracy and precision compared to FH techniques.

## Introduction

 Rehabilitation through implants has become a viable and popular option in Dentistry. Then, it is a predictable treatment option for restoring edentulous patients in addition to high predictability, which is supported by the high survival rates offered by dental implants for rehabilitation [1–3]. However, to achieve a long-term survival and success rate, multiple factors must be evaluated before placing the implants to achieve a long-term survival and success rate [4–6]. 

The clinical criteria must be always deeply observed, such as the availability and quality of hard and soft tissues, the distance between tooth-implant, and the distance between implants and critical anatomical structures (nerves, vessels, maxillary sinus, and nasal cavity), avoiding any complications correlated to the implant placement. The complications include undesired perforation, cortical or dental fenestration, and damage to specific structures due to the implant position [7, 8]. Those factors may negatively influence rehabilitation. Moreover, conventional implant placement using freehand surgery, based on two- (2D) or three-dimensional (3D) radiographic/tomographic (CBCT) evaluation, can lead to compromised accuracy or unfavorable implant placement, especially in complex cases or cases involving multiple implants. This fact can increase the short- or long-term risk of complications [9, 10]. 

Thus, new techniques have been introduced, allowing greater control and predictability in implant placement [11]. Computer-assisted implant surgery (CAIS) has been then used [12], and it includes two approaches: static (sCAIS) and dynamic (dCAIS). The CAIS’ principle is to use computed tomography (CBCT) combined with planning software that allows the virtual simulation of the implant placement pursuing the ideal three-dimensional position. It can be transferred to sCAIS and dCAIS in order to obtain clinically predictable results [13, 14]. 

Regarding sCAIS, a surgical guide is fabricated using computer-aided design and computer-aided manufacturing technology (CAD/CAM) to guide site preparation and implant placement [15–17]. However, the current literature suggests that many factors - including fixation pins, surgical guide support, manufacturing processes and materials, guide system type, and the specific drills and sleeves employed - may impact the transfer accuracy of sCAIS [18–22]. Therefore, the applicability of these findings remains uncertain, and a systematic review [13] emphasized the need for further data to evaluate potential factors influencing the accuracy of sCAIS.

On the other hand, the dCAIS system uses motion tracking technology to track the movements of the instruments, which are used to place the implants in the patient’s mandible/maxilla. This system incorporates radiopaque markers attached to the patient’s mandible/maxilla during CBCT; it will also be used during surgery, contributing to a cooperative and dynamic movement between the corresponding anatomy through the CT images and the surgical field [7]. The perception of position and movement is performed using tracking cameras set up and calibrated during the surgical procedure. It contributes to continuous tracking through sensors connected to the patient’s arch and to the surgical handpiece, displaying in real-time on the monitor superimposed on the virtual plane. Any 3D deviation of the drill and/or implant from the virtual plane can also be analyzed in real time, and the adjustment of the depth, drilling angle, or implant position can be corrected at any time. It is one of the significant advantages compared to sCAIS [23]. 

Thus, the goal of this systematic review was to assess whether dynamic navigation (dCAIS) can offer greater accuracy and precision, as well as reduced discrepancy in position, compared to guided (static) and hands-free surgery in dental implant surgery. The positive hypothesis is dCAIS has a better performance for accuracy and precision with less discrepancy compared to other systems studied.

## Materials and methods

The systematic review adhered to the guidelines outlined in the Preferred Reporting Items for Systematic Reviews and Meta-Analyses (PRISMA). The research focus question was developed using the PICO (patient, intervention, comparison, and outcome) strategy: “In partially or fully edentulous patients requiring dental implant rehabilitation, does dynamic navigation (dCAIS) provide greater accuracy, precision, and improved parallelism compared to guided surgery and freehand surgery?” Population: partially or fully edentulous patients who received dental implant placement; Intervention: navigated (dynamic) surgery; Comparison: hand-free (manual) and/or static (guided) surgery; and Outcome: effectiveness of the precision and parallelism in the implant placement among techniques. The review methods were established in advance prior to the conduct of the review, and no deviations from the original protocol were made during its execution.

### Eligibility criteria

The inclusion criteria were: (1) patients ≥ 18 years old; (2) articles published in English; (3) randomized controlled trials (RCTs), retrospective or prospective clinical studies; (4) published within the last 10 years (2014–2024). The following exclusions were applied: (1) studies older than 10 years; (2) in vitro or pre-clinical studies; (3) any review, editorial letter, or letter to the editor; and (4) studies lacking information/detail for the results; and (5) studies with unclear methodology descriptions.

### Search strategy, study selection, and data extraction

A search was carried out through PubMed/MEDLINE, Online Knowledge Library (B-On), Science Direct, Scielo, Cochrane Library, and Web of Science (between June 2024 and October 2024) using the following key terms: computer-aided surgery, dental implants, dynamic navigation, accuracy, guided-implant surgery, freehand, associated with the Boolean markers (AND and OR) (Table 1). The analysis was conducted independently by two researchers (F.C. and P.P.). In cases of doubt, a third researcher (G.V.O.F.) was assigned to analyze and resolve any ties. The inter-rater reliability level of agreement was measured using Cohen’s kappa test. First, the articles were evaluated based on their titles and abstracts. Then, the selected articles were followed for full-text reading.

**Table 1:** Search strategy per database

DATABASE	SEARCH STRATEGY
PubMed/MEDLINE	(“computer-aided surgery“[MeSH] OR “computer-aided surgery“[tiab] OR “computer assisted surgery“[tiab]) AND (“dental implants“[MeSH] OR “dental implants“[tiab] OR “dental implant“[tiab] OR implant*[tiab]) AND (“dynamic navigation“[tiab] OR “image-guided surgery“[MeSH] OR “image guided surgery“[tiab] OR “surgical navigation“[tiab]) AND (accuracy[tiab] OR accurate[tiab]) AND (“guided-implant surgery“[tiab] OR “guided implant placement“[tiab]) AND (freehand[tiab] OR “free hand“[tiab])
Online Knowledge Library (B-On)	(“computer-aided surgery” OR “computer assisted surgery”) AND (“dental implants” OR implant*) AND (“dynamic navigation” OR “image guided surgery” OR “surgical navigation”) AND (accuracy OR accurate) AND (“guided-implant surgery” OR “guided implant placement”) AND (freehand OR “free hand”)
ScienceDirect	(TITLE-ABS-KEY(“computer-aided surgery” OR “computer assisted surgery”)) AND (TITLE-ABS-KEY(“dental implants” OR implant*)) AND (TITLE-ABS-KEY(“dynamic navigation” OR “image guided surgery” OR “surgical navigation”)) AND (TITLE-ABS-KEY(accuracy OR accurate)) AND (TITLE-ABS-KEY(“guided-implant surgery” OR “guided implant placement”)) AND (TITLE-ABS-KEY(freehand OR “free hand”))
SciELO (Scientific Electronic Library Online)	(computer-aided surgery OR “computer assisted surgery”) AND (dental implants OR implant*) AND (dynamic navigation OR “guided-implant surgery”) AND accuracy AND (freehand)
Cochrane Library	#1: MeSH descriptor: [Dental Implants] #2: “dental implants”:ti, ab OR implant*:ti, ab #3: #1 OR #2 #4: MeSH descriptor: [Computer-Assisted Surgery] #5: “computer-aided surgery”:ti, ab OR “computer assisted surgery”:ti, ab OR “dynamic navigation”:ti, ab OR “image guided surgery”:ti, ab OR “surgical navigation”:ti, ab OR “guided-implant surgery”:ti, ab OR “guided implant placement”:ti, ab #6: #4 OR #5 #7: accuracy: ti, ab OR accurate: ti, ab #8: freehand: ti, ab OR “free hand”:ti, ab #9: #3 AND #6 AND #7 AND #8
Web of Science	TS=(“computer-aided surgery” OR “computer assisted surgery”) AND TS=(“dental implants” OR dental NEAR/2 implant*) AND TS=(“dynamic navigation” OR “image guided surgery” OR “surgical navigation”) AND TS=(accuracy OR accurate) AND TS=(“guided-implant surgery” OR “guided implant placement”) AND TS=(freehand OR “free hand”)

Data extraction from the selected articles focused on the author’s name, publication year, technique employed, the significance levels found from each study according to the parameters evaluated and reported outcomes (3D deviation at platform, apex level, and angulation).

### Risk of bias assessment

After reading and analyzing the included articles, the risk of bias was assessed using the Joanna Briggs Institute (JBI) Critical Appraisal tool [24, 25]. Two independent investigators (F.C. and P.P.) performed the quality assessment, and in cases of divergence, a third researcher was consulted (G.V.O.F.). Then, the risk of bias was sorted as: low risk of bias (plausible bias unlikely to alter the results seriously) if all criteria were met (all green [yes]) or at maximum two “unclear” were present; moderate risk of bias (“plausible bias” data raises some doubt about the results) if two “no” (red) was found or up to 4 “unclear” criteria were met; and high risk of bias (plausible bias that seriously weakens confidence in the results), at least 3 “no” (red) or ≥ 5 “unclear” was found.

### Meta-analysis

All statistical analyses were conducted using R (v. 4.3.1) using the meta package. A comparative analysis of study results was employed for variables where meta-analysis was not applicable. The random-effects model was used in the analysis of the variables, given the expected clinical heterogeneity in surgical protocols and measurement techniques. The heterogeneity analysis was conducted using Cochran’s Q-test and Higgins’ I^2^ statistic, with the following classifications: 0–29% indicating low heterogeneity, 30–49% indicating moderate heterogeneity, 50–75% indicating substantial heterogeneity, and 76–100% indicating considerable heterogeneity. Standardized mean differences were used as a method for measuring the effect. By dividing the mean difference of each study by its standard deviation, a comparable measure was obtained across the studies. Publication bias evaluation was performed through visual inspection of funnel plots (effect size vs. precision), Egger’s linear regression test (statistical asymmetry assessment), and contour-enhanced funnel plots to distinguish bias from heterogeneity.

## RESULTS

**Fig. 1:**
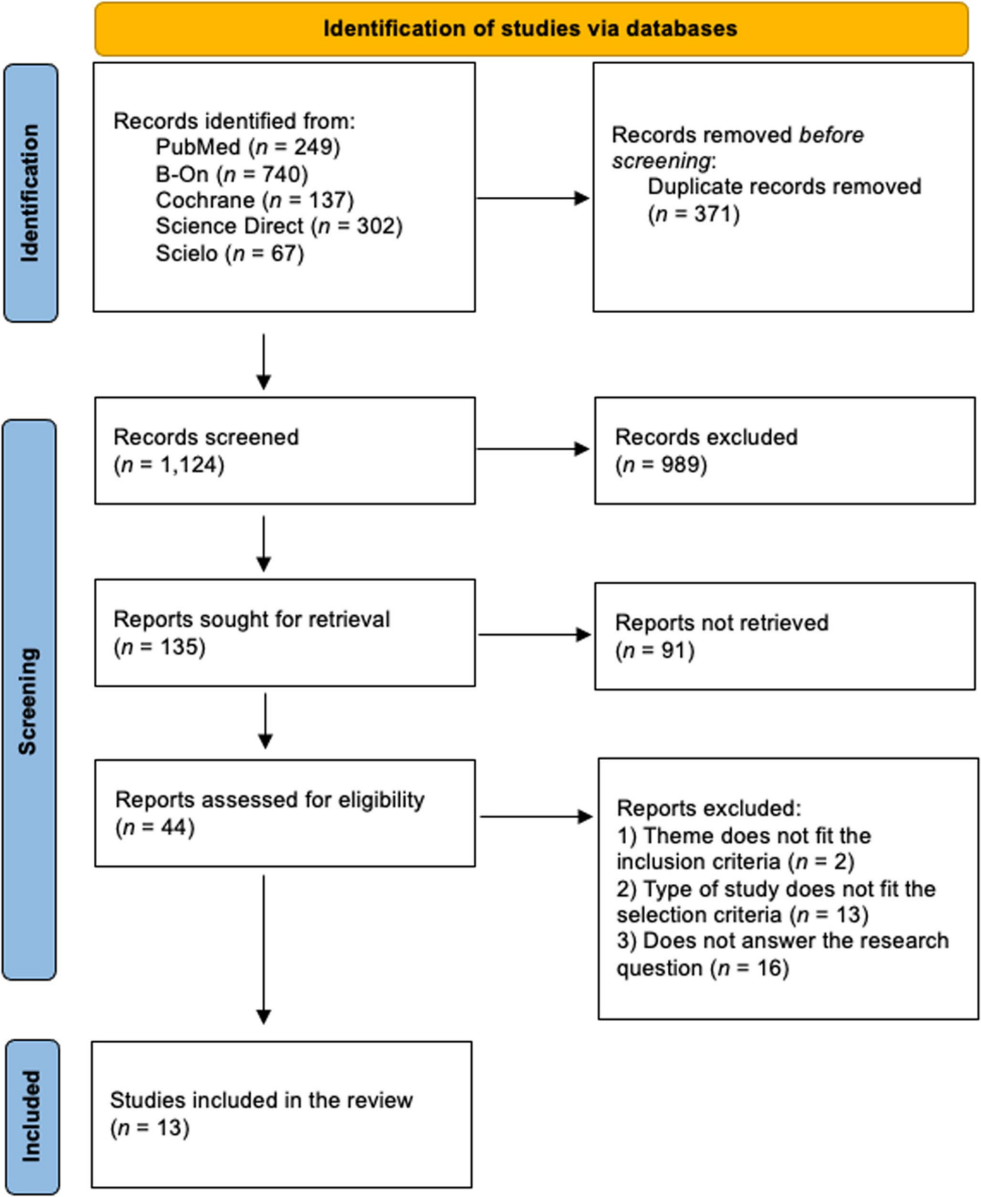
Flow diagram for selection and inclusion of articles

During the identification phase, 1,495 articles were identified, of which 371 were removed due to duplication, resulting in 1,124 articles. In the selection phase, 989 were eliminated because they were not deemed interesting based on their titles and years, and consequently, only 135 articles remained relevant. After reading the abstract, 91 articles were eliminated, leaving 44 articles that were thoroughly analyzed by the two researchers (FC and PP), and in cases of doubt, examined by a third researcher (GVOF). After reading the 44 articles in full, two articles were excluded because they did not meet the inclusion criteria, 13 articles because the type of study did not fit the selection criteria, and 16 articles because they did not answer the research question. Thus, of the 44 articles analyzed, 13 were selected [7, 26–37] that met the inclusion and exclusion criteria defined by the reviewers after full-text reading (Fig. 1).

A total of 13 studies were included: four prospective studies [26, 28, 30, 32], two retrospective studies [29, 33], and seven randomized clinical trials (RCTs) [7, 27, 31, 34–37]. Altogether, 554 patients and 687 implants were enrolled. Of these, 215 implants were placed using the freehand (FH) approach, 195 with static computer-aided implant surgery (sCAIS), and 277 with dynamic computer-aided implant surgery (dCAIS). Eight studies used dynamic CAIS, 7 used FH, and 10 approached static CAIS (two used as a pilot, one used partially guided, and 10 used full guided). Three studies compared FH and dynamic CAIS, five compared FH and static CAIS, and five compared dynamic and static CAIS.

### Accuracy and precision findings

Table 2 summarizes the outcomes and discrepancies between planned and actual implant positions across FH, sCAIS, and dCAIS.

**Table 2:** Outcomes obtained from each Article included according to the technique

Author		Dynamic Cais				Static Cais (Pilot)			
	n patients	n implants	3Ddev Plat	3Ddev Apx	3Ddev Ang	n implants	3Ddev Plat	3Ddev Apx	3Ddev Ang
Liu et al*.* (2022)	32	20	1.07 ± 0.57	1.26 ± 0.53	2.14 ± 1.20	18	-	-	-
Kaewsiri et al. (2019)	60	30	1.05 ± 0.44	1.29 ± 0.50	3.06 ± 1.37	30	-	-	-
Aydemir and Arisan (2020)	30	43	1.01 ± 0.07	1.83 ± 0.12	5.59 ± 0.39	-	-	-	-
Yimarj et al. (2020)	30	30	1.24 ± 0.39	1.58 ± 0.56	3.78 ± 1.84	30	-	-	-
Varga et al. (2020)	101	-	-	-	-	49/51/52	1.57 ± 0.91	1.86 ± 1.09	5.71 ± 3.68
Block et al. (2017)	100	80	1.37 ± 0.55	1.56 ± 0.69	3.62 ± 2.73	-	-	-	-
Smitkarn et al. (2019)	52	-	-	-	-	30	-	-	-
Kim et al. (2022)	11	-	-	-	-	24	-	-	-
Feng et al. (2022)	40	20	1.06 ± 0.55	1.18 ± 0.53	3.23 ± 1.67	20	-	-	-
Younes et al. (2018)	32	-	-	-	-	21/21	1.12 ± 0.10	1.43 ± 0.18	5.95 ± 0.87
Battista et al. (2022)	12	22	0.77 ± 0.25	1.2 ± 0.61	2.5 ± 0.41	-	-	-	-
Ayman et al. (2022)	22	-	-	-	-	11	-	-	-
Sun et al. (2020)	32	32	1.25 ± 0.09	0.73 ± 0.13	3.24 ± 0.36	32	-	-	-

Author	Static Cais (Partial)			Static Cais (Full Guided)			Free Hand			
	3Ddev Plat	3Ddev Apx	3Ddev Ang	3Ddev Plat	3Ddev Apx	3Ddev Ang	n implants	3Ddev Plat	3Ddev Apx	3Ddev Ang
Liu et al*.* (2022)	-	-	-	0.92 ± 0.46	1.31 ± 0.43	3.31 ± 1.61	-	-	-	-
Kaewsiri et al. (2019)	-	-	-	0.97 ± 0.44	1.28 ± 0.46	2.84 ± 1.71	-	-	-	-
Aydemir and Arisan (2020)	-	-	-	-	-	-	43	1.70 ± 0.13	2.51 ± 0.21	10.04 ± 0.83
Yimarj et al. (2020)	-	-	-	1.04 ± 0.67	1.54 ± 0.79	4.08 ± 1.69	-	-	-	-
Varga et al. (2020)	1.37 ± 0.79	1.59 ± 0.86	4.30 ± 3.33	1.40 ± 0.54	1.59 ± 0.59	3.04 ± 1.51	55	1.82 ± 0.94	2.43 ± 0.98	7.03 ± 3.44
Block et al. (2017)	-	-	-	-	-	-	20	1.67 ± 0.43	2.51 ± 0.86	7.69 ± 4.92
Smitkarn et al. (2019)	-	-	-	1.0 ± 0.6	1.3 ± 0.6	3.1 ± 2.3	30	1.5 ± 0.7	2.1 ± 1.0	6.9 ± 4.4
Kim et al. (2022)	-	-	-	0.97 ± 0.37	1.13 ± 0.36	3.42 ± 2.12	-	-	-	-
Feng et al. (2022)	-	-	-	0.99 ± 0.63	1.50 ± 0.75	3.07 ± 2.18	-	-	-	-
Younes et al. (2018)	-	-	-	0.73 ± 0.10	0.97 ± 0.19	2.30 ± 0.92	24	1.45 ± 0.10	2.11 ± 0.18	6.99 ± 0.87
Battista et al. (2022)	-	-	-	-	-	-	-	-	-	-
Ayman et al. (2022)	-	-	-	0.69 ± 0.36	1.26 ± 0.42	3.14 ± 1.37	11	1.43 ± 1.14	2.35 ± 1.05	3.18 ± 0.96
Sun et al. (2020)	-	-	-	1.49 ± 0.08	1.00 ± 0.15	4.54 ± 0.29	32	1.89 ± 0.09	1.42 ± 0.25	6.12 ± 0.12

Dev = deviation; Plat = platform; Ang = angle; Apx = Apex.

### sCAIS vs. dCAIS

Liu et al. [33] and Kaewsiri et al. [7] reported no significant differences between sCAIS and dCAIS in platform (Plat), apex (Apx), or angulation (Ang) deviations. Discrepancies were around 1 mm at the platform, 1.3 mm at the apex, and 2–3° in angulation. Similarly, Yimarj et al. [37] found comparable deviations for both techniques (Plat: 1.04–1.58 mm; Apx: 1.04–1.58 mm; Ang: 3.78–4.08°; *p* > 0.05).

#### dCAIS vs. FH

Aydemir and Arisan [34] demonstrated significantly smaller deviations for dCAIS (Plat: 1.01 ± 0.07 mm, Apx: 1.83 ± 0.12 mm, Ang: 5.59 ± 0.39°) compared to FH (Plat: 1.70 ± 0.13 mm, Apx: 2.51 ± 0.21 mm, Ang: 10.04 ± 0.83°; *p* < 0.001). Block et al. [28] confirmed these results, with dCAIS showing lower deviations in Plat (1.37 ± 0.55 vs. 1.67 ± 0.43 mm, *p* = 0.013) and Apx (1.56 ± 0.69 vs. 2.51 ± 0.86 mm, *p* = 0.0065). Angulation discrepancies trended lower for dCAIS (3.62 ± 2.73 vs. 7.69 ± 4.92°) but did not reach statistical significance (*p* = 0.0895).

#### sCAIS vs. FH

Varga et al. [36] reported that fully guided static protocols achieved the lowest deviations (Plat: 1.40 ± 0.54 mm, Apx: 1.59 ± 0.59 mm, Ang: 3.04 ± 1.51°), significantly outperforming FH (Plat: 1.82 ± 0.94 mm, Apx: 2.43 ± 0.98 mm, Ang: 7.03 ± 3.44°; *p* < 0.05). Similarly, Smitkarn et al. [28] found significantly greater accuracy with sCAIS compared to FH (Plat: 1.0 ± 0.6 vs. 1.5 ± 0.7 mm; Apx: 1.3 ± 0.6 vs. 2.1 ± 1.0 mm; Ang: 3.1 ± 2.3 vs. 6.9 ± 4.4°; all *p* = 0.001). Ayman et al. [35] noted that sCAIS significantly reduced apex discrepancies versus FH (1.26 ± 0.42 vs. 2.35 ± 1.05 mm, *p* = 0.042), though platform and angulation differences were not significant. Younes et al. [31] confirmed that fully guided (FG) protocols performed best, with minimal deviations (Plat: 0.73 ± 0.10 mm, Apx: 0.97 ± 0.19 mm, Ang: 2.30 ± 0.92°), surpassing both FH and pilot-drill (Pi) protocols.

#### Clinical acceptability and esthetic zone

Battista et al. [30] demonstrated that dCAIS achieved high precision in esthetic-zone implants (Plat: 0.77 ± 0.25 mm, Apx: 1.2 ± 0.61 mm, Ang: 2.5 ± 0.41°). Kim et al. [26] and Feng et al. [32] further confirmed that both sCAIS and dCAIS produce clinically acceptable outcomes, with deviations of approximately 1 mm (Plat/Apx) and < 4° (Ang).

#### Direct three-way comparisons

Sun et al. [29] directly compared all three methods (dCAIS, sCAIS, and FH). Significant differences were found across all parameters (*p* < 0.0001). dCAIS achieved the smallest deviations, particularly at the apex (0.73 ± 0.13 mm), followed by sCAIS (1.00 ± 0.15 mm), while FH consistently yielded the largest deviations (Apx: 1.42 ± 0.25 mm; Ang: 6.12 ± 0.12°).

### Overall trends

Across studies, guided techniques (dCAIS and fully guided sCAIS) consistently outperformed FH, with dCAIS often showing a slight advantage in apex accuracy, while sCAIS performed very well in angulation stability (Table 3).

**Table 3:** A significant level (**p**) was found in the included studies

Author	Technique	Platform (Plat)	Apex (Apx)	Angle (Ang)
Liu et al. (2022)	dCais vs. sCais	0.669	0.566	0.893
Kaewsiri et al. (2019)	dCais vs. sCais	0.47	0.94	0.60
Aydemir and Arisan (2019)	dCais vs. FH	0.001***	0.001***	0.001***
Yimarj et al. (2020)	dCais vs. sCais	0.11	0.57	0.64
Varga et al. (2020)	FH vs. Pi	0.5059	0.1054	0.1937
	FH vs. PG	0.0629	0.0012***	0.0001***
	FH vs. FG	0.2450	0.0055	0.0001***
	Pi vs. PG	0.7096	0.4654	0.1037
	Pi vs. FG	0.9758	0.7651	0.003**
	PG vs. FG	0.9016	0.9532	0.2115
Block et al. (2017)	dCais vs. FH	0.013**	0.0065**	0.0895
Smitkarn et al. (2019)	sCais vs. FH	0.001***	0.001***	0.001***
Kim et al. (2022)	sCais	0.13	0.13	NR
Feng et al. (2022)	dCais vs. sCais	0.659	0.231	0.547
Younes et al. (2018)	FH vs. Pi	0.068*	0.837	1.000
	Pi vs. FG	0.033*	0.267	0.022*
	FH vs. FG	0.001***	0.001***	0.002**
Battista et al. (2022)	dCais	NR	NR	NR
Ayman et al. (2022)	sCais vs. FH	0.0530	0.0042**	0.9462
Sun et al. (2020)	dCais vs. FG vs. FH	0.0001***	0.0001***	0.0001***

* *p*<0.05 if CI=95%; ** p≤0.01 if CI=95%; *** *p*≤0.001 if CI=95%; NR = not reported; FH = free hand; sCAIS = static computer-aided implant surgery, dCAIS = dynamic navigation, Pi = Pilot-Drill Guided Surgery, FG = Full Guided Surgery, PG = Partial Guide Surgery.

### Risk of bias and adverse events

The overall risk of bias was rated as low across all included studies (Table 4). However, none of the studies reported on adverse events or unanticipated harms, with all scoring negative on the question: “*Were adverse events (harms) or unanticipated events identified and described?*”

**Table 4:** Risk of bias of the studies included

	Liu et al. (2022)	Kaewsiri et al. (2019)	Aydemir & Ihsan (2020)	Yimarj et al. (2020)	Varga et al. (2020)	Block et al. (2017)	Smitkarn et al. (2019)	Kim et al. (2022)	Feng et al. (2022)	Younes et al. (2018)	Battista et al. (2022)	Ayman et al. (2022)	Sun et al. (2020)
Were patient’s demographic characteristic(s) clearly described?	Yes	Yes	Yes	Yes	Yes	Yes	Yes	Yes	Yes	Yes	Yes	Yes	Yes
Was the patient’s history clearly described and presented as a timeline?	Yes	Yes	Yes	Yes	Yes	Yes	Yes	Yes	Yes	Yes	Yes	Yes	Yes
Was the current clinical condition of the patient on presentation clearly described?	Yes	Yes	Yes	Yes	Yes	Yes	Yes	Yes	Yes	Yes	Yes	Yes	Yes
Were diagnostic test or assessment methods and the results clearly described?	Yes	Yes	Yes	Yes	Yes	Yes	Yes	Yes	Yes	Yes	Yes	Yes	Yes
Was the intervention(s) or treatment procedure(s) clearly described?	Yes	Yes	Yes	Yes	Yes	Yes	Yes	Yes	Yes	Yes	Yes	Yes	Yes
Was the post-intervention clinical condition clearly described?	Yes	Yes	Yes	Yes	Yes	Yes	Yes	Yes	Yes	Yes	Yes	Yes	Yes
Were adverse events (harms) or unanticipated events identified and described?	Yes	Unclear	No	No	No	No	No	No	No	No	No	No	No
Does the case report provide takeaway lessons?	Yes	Yes	Yes	Yes	Yes	Yes	Yes	Yes	Yes	Yes	Yes	Yes	Yes
RESULT	Low	Low	Low	Low	Low	Low	Low	Low	Low	Low	Low	Low	Low

Low risk of bias - all criteria were met (all green [Yes]) or at maximum two “Unclear” were present; Moderate risk of bias - two “No” (red) was found or up to 4 “unclear” criteria were met; and High risk of bias - 3 “No” (red) or ≥ 5 “Unclear” was found.

### Meta-analysis

**Fig. 2:**
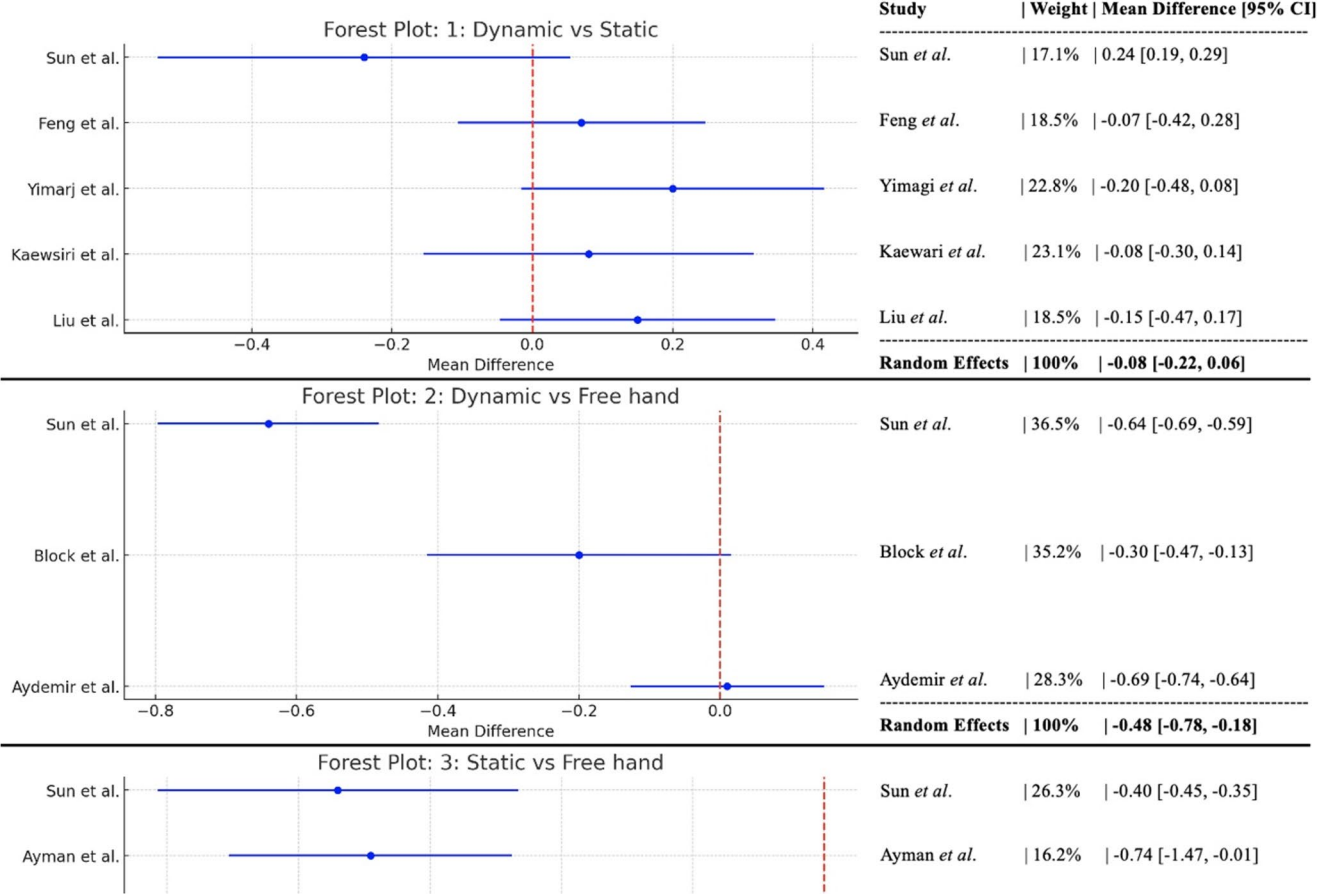
Forest plot evaluating and comparing the three groups studied

The meta-analysis compared three surgical approaches (Fig. 2): dCAIS, sCAIS, and FH. The analysis focuses on accuracy outcomes (reported as deviation results in millimeters) across multiple studies. Three primary comparisons were made: (1) dCAIS vs. sCAIS; (2) dCAIS vs. FH; (3) sCAIS vs. FH. The results for the first group were compared across five studies (*n* = 132 Dynamic, *n* = 130 Static). The pooled mean difference was − 0.08 mm (95% CI: −0.22 to 0.06), with a substantial heterogeneity (I² = 52%) and no statistically significant difference in accuracy between the Dynamic and Static CAIS approaches (*p* = 0.08).

The second comparison involved three studies (*n* = 155 Dynamic, *n* = 95 Free Hand). The pooled mean difference was − 0.48 mm (95% CI: −0.78 to −0.18), indicating considerable heterogeneity with I² = 89% and a statistically significant better accuracy for dCAIS compared to the FH approach (*p* < 0.01).

The last comparison was between sCAIS and FH. Five studies were analyzed (*n* = 146 Static, *n* = 152 Free Hand). The pooled mean difference was − 0.62 mm (95% CI: −0.90 to −0.34), with a considerable heterogeneity (I² = 84%). sCAIS showed statistically significantly better accuracy than the FH approach (*p* < 0.01).

**Fig. 3:**
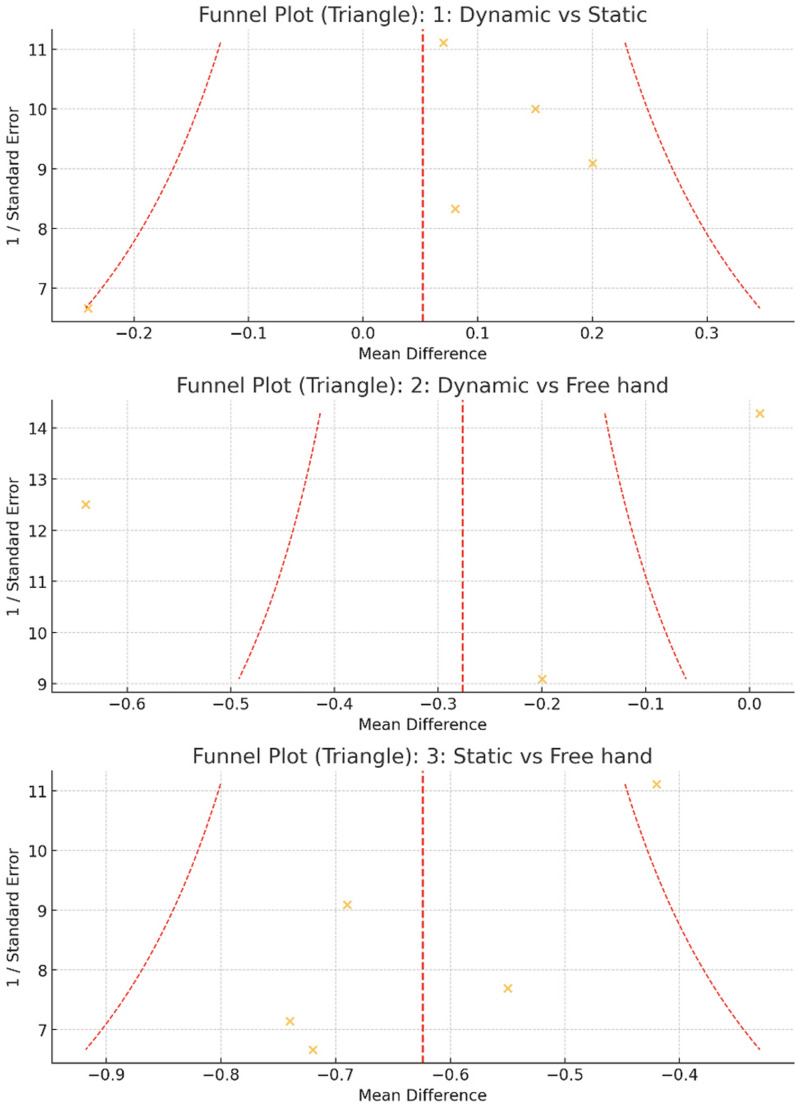
Funnel plots for all three comparisons: Dynamic Computer-Assisted Implant Surgery (Dynamic CAIS), Static CAIS (Pilot), and Free-Hand technique

Funnel plots for all three comparisons showed generally symmetrical distributions, with Egger’s tests indicating no significant publication bias (*p* > 0.05 for all comparisons) (Fig. 3). The figure displays three funnel plots, each representing one of the pairwise comparisons in the meta-analysis of accuracy for dental implant placements using dCais, sCais, and FH methods. The first plot illustrates the mean differences centered around the random-effects summary mean (red dashed line); the data points are distributed on both sides of this central line, forming an approximate funnel shape. The two red dashed curves outline the expected 95% confidence region. This symmetric distribution suggests minimal publication bias for this comparison.

The second plot similarly displays the distribution of effect sizes and standard errors for studies comparing dCais and FH methods; the data points appeared outside the expected 95% confidence interval region (red dashed lines), suggesting the data for this comparison may exhibit some degree of publication bias or small-study effects. The asymmetry (with points outside the funnel area) may also indicate heterogeneity in the results across studies, or differences in methodology or reporting.

The third plot shows the data for the comparison between sCais and FH; the data points are again well-distributed around the summary mean, maintaining the expected funnel shape. This pattern supports the assumption of no significant publication bias in this dataset.

The findings showed no significant difference between dCAIS and sCAIS approaches in terms of accuracy (mean difference − 0.08 mm, *p* = 0.26); both CAIS approaches showed significantly better accuracy than FH placement (dCAIS: 0.48 mm more accurate than FH [*p* = 0.002] and sCAIS: 0.62 mm more accurate than FH [*p* < 0.001]). Therefore, for the clinical implications, the choice between dynamic and static CAIS may depend on other factors (cost, workflow, surgeon preference), as accuracy differences are minimal. However, all data must be carefully analyzed due to the high heterogeneity in some comparisons, variation in measurement protocols across studies, and a limited number of studies for comparisons.

## Discussion

This meta-analysis aimed to verify whether dCAIS can offer greater accuracy and reduced discrepancy in position, compared to guided (static) and hands-free surgery. In comparative studies of implant placement techniques, dCAIS and sCAIS have been investigated thoroughly. Liu et al. [33] observed that dCAIS resulted in greater platform-level deviations (1.07 ± 0.57 mm) compared to sCAIS (0.92 ± 0.46 mm) in their analysis, which complements findings by Khaohoen et al. [38], who corroborated the variations in accuracy between these methodologies. Nevertheless, contrasting evidence was provided by Sun et al. [29], indicating that dCAIS could yield superior accuracy (1.25 ± 0.09 mm) at the platform level over sCAIS (1.49 ± 0.08 mm), potentially arising from differences in operator training and experience, which can greatly influence surgical outcomes [27, 31].

At the apex level, the observations by Liu et al. [33] also showed higher deviations in sCAIS (1.31 ± 0.43 mm) compared to dCAIS (1.26 ± 0.53 mm), a finding supported by other authors [27, 29, 38]. Conversely, Kaewsiri et al. [7] and Younes et al. [31] noted improved precision with sCAIS, again highlighting the variability based on surgical proficiency and the complexity of cases handled, which implies the substantial role that clinician competence plays in the accuracy of outcomes in both surgical approaches.

Regarding angulation control, the advantages of dCAIS have been acknowledged, with Liu et al. [33] reporting deviations of 2.14 ± 1.20° compared to 3.31 ± 1.61° with sCAIS. Additionally, Younes et al. noted similar trends in angular deviations (3.78 ± 1.84° for dCAIS vs. 4.08 ± 1.69° for sCAIS), while Smitkarn et al. [27] and Feng et al. [32] found situations where sCAIS demonstrated better angular accuracy, again suggesting that results may be highly dependent on the specific circumstances of each surgical practice and the proficiency of the users [27, 36].

The discussion surrounding dCAIS in comparison to FH surgical techniques reveals significant advantages of dCAIS across multiple parameters. Aydemir & Arisan [34], along with Block et al. [28], validated that dCAIS outperforms FH in terms of accuracy due to capabilities in real-time tracking and three-dimensional guidance [39]. In affirming this, Sun et al. [29] the most considerable deviations occurred with FH methods, particularly in angular measurements (6.12 ± 0.12°), thus supporting the narrative that CAIS methods enhance precision significantly over traditional techniques [31, 40].

Furthermore, when comparing static methods with FH, various RCTs [27, 31, 36] have consistently shown that sCAIS achieves better accuracy than FH. Notably, Varga et al. [36] observed that full guidance yields optimal results, although discrepancies at the platform level remained statistically insignificant (*p* = 0.9016) [31, 36, 41].

It is evident that dCAIS generally excels in angulation management while sCAIS consistently ensures more precise placements when compared to FH techniques. The influence of the operator’s expertise significantly affects outcomes across all methodologies, reinforcing the importance of training and experience within clinical settings. This highlights the need for standardized research approaches to control for confounding variables and enhance the generalizability of findings across diverse clinical environments. Thus, operator skill and learning curve critically influence outcomes [29]; real-time feedback in dCAIS reduces human error, while sCAIS relies on pre-operative guide stability.

### Strengths and limitations

As strength, it is possible to consider the comparative breadth; this review included 13 studies (prospective, retrospective, and RCTs) with a total of 554 patients and 687 implants, allowing comparisons across all three major surgical techniques (FH, sCAIS, dCAIS). Also, the diverse study designs included, RCTs alongside prospective and retrospective cohorts, which strengthens external validity while still enabling evaluation of randomized evidence. A comprehensive analysis of outcomes, presenting deviations at platform, apex, and angulation which were consistently reported, permitting a detailed comparison of accuracy and precision among techniques. The clinical applicability of the subject studied, with studies evaluated esthetic zone implants, confirming that guided approaches (particularly dCAIS) maintain accuracy in challenging areas. Consistency of findings, that despite different designs and settings, the majority of studies converged on the superiority of guided protocols (especially dCAIS and fully guided sCAIS) compared to FH.

As limitations, the heterogeneity of methodologies, with variability existed in implant systems, imaging modalities, surgical protocols (fully guided vs. pilot/partial), and measurement methods, limiting the possibility of direct quantitative synthesis (meta-analysis); the sample size variability, in which some included studies had relatively small sample sizes, reducing statistical power and potentially limiting generalizability. The risk of bias in reporting should be mentioned; although overall risk of bias was low, all studies failed to report on adverse events or complications, which may underestimate potential risks of guided navigation. The short-term evaluation, with most studies assessed deviations at the time of implant placement without long-term follow-up, leaving questions about the impact of surgical accuracy on clinical outcomes (osseointegration, prosthetic success, survival rates). Operator dependency, due to differences in clinician experience with navigation and static guides were not consistently addressed, which may influence accuracy outcomes. Limited direct comparisons, with only a few studies directly compared all three methods (FH, sCAIS, and dCAIS) in the same design, restricting strong head-to-head conclusions.

## Conclusion

According to the studies evaluated, considering the study’s limitations, this meta-analysis confirms that CAIS, whether dCAIS or sCAIS, significantly improves accuracy compared to the FH approach. While dCAIS showed a slight numerical edge over sCAIS, the difference was not statistically significant, leading to the rejection of the hypothesis that dCAIS outperforms sCAIS and FH. The choice between dCAIS and sCAIS should consider factors such as clinical context, operator experience, and equipment availability, as both methods demonstrate superior outcomes over FH. Nonetheless, dynamic navigation, despite its flexibility, presents challenges such as higher costs and a steep learning curve. Future standardized studies with larger samples and long-term follow-up are needed further to clarify the comparative effectiveness and precision of these techniques.

## Data Availability

No datasets were generated or analysed during the current study.
